# Supplementary material for: The Production of Helianthus Haploids: A Review of Its Current Status and Future Prospects
Source: Plants (Basel). 2022 Oct 29;11(21):2919. doi: 10.3390/plants11212919 (PMC9654405; doi:10.3390/plants11212919)
Supplement: Supplementary file 1 [file plants-11-02919-s001.zip › plants-1911895-supplementary.pdf]

Table S1: Similarities and differences in the structural organization of formed sunflower zygotic and gynogenic haploids (according to [82]).

Trait/organelle	Zygotic embryo	Gynogenic embryo
**Zygote/activated ovule**		
Time moment of ovule activation	Several hours after fertilization	Several days after the start of *in vitro* ovule cultivation
Structural organization of organelles	Increase in the number and metabolic activity of organelles during development: numerous plastids localized to the periphery and mitochondria with well-developed cristae; increase in the Golgi apparatus and endoplasmic reticulum (ER). Decrease in the density of the ribosomes situated near the periphery of nucleus, on the outer nuclear membrane, and in the cytoplasm with maturation. Migration of the nucleus to the center from its typical position near the chalazal pole	
Number of lipid inclusions	Lower	Higher
Vacuoles	Numerous small vacuoles in the micropyle end, demonstrating the presence of polarity	Increased in size and more evenly distributed in the cytoplasm, suggesting a decrease in the initial polarity or its disappearance
Cell wall	Zygote divides *in vivo* before the cell wall is formed at the chalazal end	During the cultivation, the cell wall completes its formation around the egg cell (including the chalazal end) before the first division
**Proembryo**		
Structural organization of organelles	High density of ribosomes and mitochondria; diversity of Golgi bodies with numerous bubbles, well-developed ER; and disappearance of lipid droplets	
Development rate and uniformity	More rapid and more uniform	Slower and less uniform; some nuclei are subject to free division giving coenocytes
Vacuoles	Lower number of vacuoles; autophagic vacuoles are formed only in the suspensor	Larger number of vacuoles; autophagic vacuoles are frequently formed in embryoid and suspensor
Position of embryoid in embryo sac	Suspensor is directed towards the micropyle end and embryoid cells, to the chalazal-end; this is most likely associated with the uptake of nutrients through the chalazal end	Electron dense cells in the micropyle end give rise to embryoid; electron light cells resembling suspensor reside in the chalazal end. Embryos with normal polarity are developed; this may be associated with the uptake of nutrients through the micropyle end
Cell walls	Thicker cell walls;<br>formed through the phragmoplast immediately after each mitosis	Thinner cell walls;<br>formed in a fragmentary manner; their centripetal growth is observed
